# AIDS Vaccine Research Subcommittee (AVRS) Consultation: Early-Life Immunization Strategies against HIV Acquisition

**DOI:** 10.1128/mSphere.00320-19

**Published:** 2019-07-17

**Authors:** Anjali Singh, Sallie Permar, Tobias R. Kollmann, Ofer Levy, Mary Marovich, Kristina De Paris

**Affiliations:** a Vaccine Research Program, Division of AIDS, National Institute of Allergy and Infectious Diseases, National Institutes of Health, Rockville, Maryland, USA; b Department of Pediatrics, Duke University Medical School, Durham, North Carolina, USA; c Human Vaccine Institute, Duke University Medical School, Durham, North Carolina, USA; d Department of Pediatrics, Division of Infectious Diseases, University of British Columbia, Vancouver, British Columbia, Canada; e Precision Vaccines Program, Division of Infectious Diseases, Boston Children’s Hospital, Boston, Massachusetts, USA; f Harvard Medical School, Boston, Massachusetts, USA; g Broad Institute of MIT and Harvard University, Cambridge, Massachusetts, USA; h Department of Microbiology and Immunology, University of North Carolina, Chapel Hill, North Carolina, USA; University of Pittsburgh School of Medicine

**Keywords:** HIV, broadly neutralizing antibody, early life, immune ontogeny, vaccine

## Abstract

This report summarizes a consultation meeting convened by the National Institute of Allergy and Infectious Diseases (NIAID), National Institutes of Health (NIH), on 12 September 2017 to discuss the scientific rationale for selectively testing relevant HIV vaccine candidates in early life that are designed to initiate immune responses for lifelong protective immunity. The urgent need to develop interventions providing durable protective immunity to HIV before sexual debut coupled with the practicality of infant vaccine schedules supports optimizing infant HIV vaccines as a high priority. The panelists discussed the unique opportunities and challenges of testing candidate HIV vaccines in the context of distinct early-life immunity. Key developments providing rationale and grounds for cautious optimism regarding evaluation of early-life HIV vaccines include recent studies of early-life immune ontogeny, studies of HIV-infected infants demonstrating relatively rapid generation of broadly neutralizing antibodies (bNAbs), discovery of novel adjuvants active in early life, and cutting-edge sample-sparing systems biology and immunologic assays promising deep insight into vaccine action in infants. Multidisciplinary efforts toward the goal of an infant HIV vaccine are under way and should be nurtured and amplified.

**IMPORTANCE** Young adults represent one of the highest-risk groups for new HIV infections and the only group in which morbidity continues to increase. Therefore, an HIV vaccine to prevent HIV acquisition in adolescence is a top priority. The introduction of any vaccine during adolescence is challenging. This meeting discussed the opportunities and challenges of testing HIV vaccine candidates in the context of the infant immune system given recent advances in our knowledge of immune ontogeny and adjuvant design and studies demonstrating that HIV-infected infants generate broadly neutralizing antibodies, a main target of HIV vaccines, more rapidly than adults. Considering the global success of pediatric vaccines, the concept of an HIV vaccine introduced in early life holds merit and warrants testing.

## Abstract (precis)

Young adults represent one of the highest-risk groups for new HIV infections and the only group in which morbidity continues to increase. Therefore, an HIV vaccine to prevent HIV acquisition in adolescence is a top priority. The introduction of any vaccine during adolescence is challenging. This meeting discussed the opportunities and challenges of testing HIV vaccine candidates in the context of the infant immune system given recent advances in our knowledge of immune ontogeny and adjuvant design and studies demonstrating that HIV-infected infants generate broadly neutralizing antibodies, a main target of HIV vaccines, more rapidly than adults. Considering the global success of pediatric vaccines, the concept of an HIV vaccine introduced in early life holds merit and warrants testing.

## EARLY-LIFE IMMUNIZATION AGAINST HIV

The implementation of pediatric vaccines against multiple infectious diseases, supported by the WHO Expanded Program on Immunization (EPI), markedly benefits global health by significantly decreasing childhood mortality and morbidity (1, 2). Major progress toward reducing pediatric AIDS has been achieved mainly by scaling up HIV treatment and prevention programs in women, but as the number of children and youth becoming newly infected with HIV remains unacceptably high (3), especially in young women who can become pregnant, efforts to expedite the discovery, manufacture, and testing of pediatric vaccines for HIV need to be more strongly emphasized. This goal is aligned with the UNAIDS Start Free-Stay Free-AIDS Free collaborative framework that embraces a life cycle approach to achieve an AIDS-free generation with a particular focus on children, adolescents, and young women (4).

A consultation committee, in conjunction with the NIH AIDS Vaccine Research Subcommittee (AVRS) advisory panel meeting in September 2017, solicited suggestions about the critical gaps in the current research agenda related to early-life vaccination and the need to develop organized efforts, such as pediatric vaccine working groups, to address the prioritized gap areas relevant to pediatric HIV vaccine research and development.

The meeting was organized into three sessions. The first session was entitled “Pediatric Immune Landscape: Immune Ontogeny and Responses to Vaccines” and was moderated by Barton F. Haynes (Duke University School of Medicine, Durham, NC) and Mary Marovich (National Institutes of Health, Bethesda, MD). The objective of this session was to understand the distinct attributes of the infant immune system and how to exploit these features to develop effective HIV vaccine strategies for eliciting optimal immune responses in infants and, potentially, achieving lifelong immunity. Glenda Gray (University of the Witwatersrand, South Africa) and Sharon Nachman (Stony Brook University, Stony Brook, NY) comoderated the discussion “Pediatric HIV Vaccination—Past Trials and Current Plans,” which aimed to highlight how prior vaccine trials in infants may inform further and fine tune the deliverables for the ongoing and planned infant HIV vaccine clinical trials. This session naturally led to the final round of talks, “Pediatric HIV Vaccines—Clinical Candidates and Immunization Strategies,” which were comoderated by John R. Mascola (National Institutes of Health, Bethesda, MD) and Jean Patterson (National Institutes of Health, Bethesda, MD). The focus of the session was to review promising HIV immunogen platforms that could be tested in infants for elicitation of protective immunity and to discuss how initiating HIV vaccination in infancy may allow long-term maturation of vaccine-elicited immune responses over time, which could then be boosted in adolescence for achieving highly mature and broad immunity. The interactive atmosphere led to frequent cross-references of the questions addressed by the various speakers in the different sessions, with the key points being presented here.

## PEDIATRIC IMMUNE LANDSCAPE: IMMUNE ONTOGENY AND RESPONSES TO VACCINES

John E. Sleasman (Duke University School of Medicine, Durham, NC) opened this session with a review of normal B cell and antibody development in healthy, HIV-unexposed human infants. He emphasized that early life is a highly unique and rapidly changing immunological landscape that may provide particular advantages on the cellular level for the induction of protective HIV immune responses following vaccination. Specifically, Dr. Sleasman pointed out that the high germinal center B cell activity and lower frequency of B10 regulatory cells in early life may provide an ideal opportunity to enhance infant B cell priming following HIV vaccination. This was consistent with findings by Dr. Haynes in infant nonhuman primates (NHPs), where expression profiles of marginal zone B cells revealed a high level of activation at baseline. Dr. Haynes noted that marginal zone B cells in NHPs are enriched with autoreactive/polyreactive cells, raising the possibility that self-reactive B cells may be required to develop broadly neutralizing antibodies (bNAbs). In addition, or alternatively, Dr. Sleasman stated that the relatively high levels of somatic hypermutation (SHM)-negative sequences in IgA and IgG classes in the first year of life, coupled with the continued terminal transferase activity after birth, are key to Ig heavy-chain repertoire plasticity and could support the selection of bNAbs.

Dr. Haynes further presented a comparison of HIV Env responses to vaccination of infant and adult rhesus macaques to (i) a DNA prime/recombinant adenovirus 5 (rAd5) trivalent A, B, C gp140CF boost regimen, the NIH Vaccine Research Center (VRC) vaccine used in the HVTN505 efficacy trial, and (ii) immunization with CH505 SOSIPs, HIV envelope trimers stabilized by disulfide bonds (SOS) between gp120 and gp41 and an additional mutation of isoleucine to proline (IP) at position 559. The researchers found no evidence of reduced immunogenicity following early-life HIV-specific immunization; tier 1 NAbs were of similar magnitudes in infant and adult rhesus macaques. However, the immunization protocols did not induce bNAbs, precluding any conclusions about the effect of age on bNAb induction by HIV vaccination in NHPs.

These preclinical vaccinology data were contrasted by findings presented by Maximilian Muenchhoff (University of Oxford, Oxford, United Kingdom) in human HIV-infected pediatric nonprogressors, defined as vertically infected children who remain clinically asymptomatic and maintain normal CD4 peripheral blood cell counts despite persistently high viremia. These infants have both HIV-specific T and B cell responses that increase with age (5). Interestingly, the HIV-infected pediatric nonprogressors also develop potent bNAb responses that are associated with T follicular helper cell phenotype and function (5). Similarly, Julie Overbaugh (Fred Hutchinson Cancer Research Center, Seattle, WA) and colleagues detected bNAbs in vertically infected infants (6, 7). In contrast to HIV-infected adults who generally present with a specific and dominant bNAb response, HIV-infected infants exhibited polyclonal bNAb responses and/or had bNAbs directed to novel epitopes (6, 7). Dr. Overbaugh emphasized that persistence of high viral loads, as observed in her studies and those of Dr. Muenchhoff, likely are required for the selection and maturation of bNAbs, an aspect that is not readily recapitulated by vaccination. Dr. Overbaugh further stressed that HIV infection in infants, intrapartum or via breastfeeding, occurs despite passively acquired HIV-specific antibodies from the mother and that maternal antibodies can impact the *de novo* formation of infant antibody responses. Of note, monoclonal bNAbs from HIV-infected infants have relatively low SHM, and although they can bind and effectively neutralize autologous virus, they do not neutralize the transmitted virus from the mother. These results may be highly relevant as it is these neutralization escape variants that are transmitted from mother to infant.

Overall, the data reviewed clearly indicate that bNAbs against HIV can be generated in early life (5–7); successful induction of bNAbs may relate not only to the distinct immune landscape of early life but also to factors external to the infant, such as persistence of high antigen load, as well as immune modulation via maternal antibodies.

In the final part of this session, Arnaud Marchant (Universite Libre de Bruxelles, Brussels, Belgium) discussed findings relevant to immune response to vaccines in HIV-exposed, uninfected (HEU) infants. The immune landscape of HEU infants is in fact very different from that of HIV-unexposed and -uninfected (HUU) infants. Therefore, HIV vaccine trials in HUU infants may find distinct mechanisms at play compared to those in the HEU infant. While these distinct infant populations could be considered an obstacle to early-life vaccination, Tobias Kollmann (University of British Columbia, Vancouver, Canada) argued that it is precisely the differences in immune response to vaccination between groups and individuals that will allow identification of mechanisms resulting in protective immunity. Dr. Kollmann then presented a protocol for the processing of small volumes (≤1 ml) of infant blood samples for multi-omic assays, thereby enabling study of systems vaccinology in early life (8). This “small sample, big data” protocol was developed in collaboration with Ofer Levy (Precision Vaccines Program, Boston Children’s Hospital, Boston, MA), who summarized current knowledge regarding age-specific vaccine adjuvant activity in early life. Dr. Levy emphasized that the activity of adjuvants varies with age and that such differences can be accurately modeled using human *in vitro* assay systems (9), identifying, for example, TLR7/8 and STING agonists that induce a balanced Th1/Th2 cytokine profile in early life *in vivo* (10, 11). Such age-specific adjuvantation strategies may enhance immunogenicity of early-life HIV vaccines. Georgia D. Tomaras (Duke University School of Medicine, Durham, NC) complemented these statements and built upon the theme of sample-sparing assays, describing those that enable analysis of antibody responses. Several of these approaches, including linear epitope array mapping and binding antibody multiplex assays, have already been successfully used in pediatric studies (12–14).

## PEDIATRIC HIV VACCINATION—PAST TRIALS AND CURRENT PLANS

Elizabeth J. McFarland (Children’s Hospital Colorado, Aurora, CO) reviewed previous pediatric HIV vaccine trials, that, although few in number, have demonstrated the feasibility of inducing HIV-specific immune responses in early infancy by vaccination. The PACTG 230 trial compared two HIV envelope vaccines, the VaxGen recombinant (r) gp120 MN with aluminum hydroxide as adjuvant and the rgp120-SF2 developed by Chiron and adjuvanted with MF59 in newborns of HIV-infected mothers. Despite the limited numbers of participants in each study arm, the trial aimed to be comprehensive in evaluating key vaccine parameters, including different vaccine doses and immunization intervals. Important for a pediatric vaccine to prevent breast milk transmission of HIV, superior gp120-specific antibody responses were induced by lower doses of gp120, by an accelerated interval (weeks 0, 2, and 8), and by incorporating MF59 instead of alum as adjuvant (15–17). This study was followed by the PACTG 236 trial that evaluated the safety and immunogenicity of recombinant canarypox virus (ALVAC)-HIV vaccines in an effort to enhance cytotoxic T cell and antibody responses in infants born to HIV-infected women. While the ALVACvCP205 vaccine induced cytotoxic T lymphocyte (CTL) responses in about 50% of infants, antibody responses induced by active immunization could not be accurately measured due to the presence of maternal antibodies. In contrast, an ALVACvCP1452 prime/rgp120 protein (alum adjuvant) boost regimen induced persistent antibody responses in infants (18, 19). Thus, these early pediatric HIV vaccine trials clearly demonstrated that HIV vaccines can induce Env-specific antibody responses in infants, including HEU infants, and that these responses are not inhibited by maternal antibodies. Importantly, a retrospective analysis of plasma samples from the PACTG 230 trial demonstrated that antibody responses persisted for close to 2 years, unlike in adults, and the frequencies of V1V2 responses at peak were similar to or even exceeded those observed in adults receiving a similar rgp120 vaccine in the RV144 HIV vaccine trial in adults (20–22).

Dr. McFarland reminded the forum that despite these encouraging vaccinology results, pediatric HIV prevention strategies are focused predominantly on early intervention and passive prevention methods, strategies that are equally important but address distinct infection scenarios and do not obviate a pediatric HIV vaccine. Currently, two important studies are ongoing. The case of the Mississippi baby (23, 24) instilled the hope that very early antiretroviral therapy (ART) initiation in infants diagnosed at birth can significantly limit the establishment of a viral reservoir, potentially resulting in remission, or at a minimum, open the door for successful future functional cure strategies in the pediatric population. To test this exciting idea, the IMPAACT trial P1115 (NCT02140255) began in 2014 and includes sites in the United States, sub-Saharan Africa, Southeast Asia, and South America. Second, to address the problem of breast milk transmission of HIV, which now accounts for the majority of new pediatric HIV infections, the IMPAACT P1112 trial (NCT02256631) started in the summer of 2017. The goal of this study is to test the safety and pharmacokinetics (PK) of the bNAb VRC01 (25–28) in newborns. Importantly, the P1112 trial will also include the evaluation of the long-lasting version of VRC01LS (29). If the treatment is proven safe and has acceptable PK parameters, it is possible that in the foreseeable future, HIV-exposed infants could receive passive immunizations with cocktails of bNAbs at just a few time points throughout their breastfeeding period. However, significant obstacles remain to achieve this goal, including a scientifically sound rationale as to which antibodies should be included in this passive immunization strategy to achieve the breadth of coverage needed to prevent HIV transmission by breast milk in infants worldwide.

Kristina De Paris (University of North Carolina, Chapel Hill, NC) emphasized that the potential of passive immunization strategies in preventing breast milk transmission was documented almost 20 years ago in NHP studies. The transfer of plasma from simian immunodeficiency virus (SIV)-infected rhesus macaques to newborns was protective against high-dose oral SIV challenge in infant macaques (30), and several studies since then have confirmed that antibodies present at the time of infection can prevent oral SIV acquisition (31, 32). Referring back to the IMPAACT P1112 trial, Dr. De Paris highlighted the studies by Hessell et al. from the Haigwood group in which they could demonstrate that passive administration of VRC01 to infant rhesus macaques can protect against oral SHIV acquisition (33), providing proof of concept for the IMPAACT P1112 trial in human infants.

Dr. De Paris further asserted that these promising prevention tools in the fight against pediatric HIV infection should not diminish our quest for developing an HIV vaccine for long-term protection. She described challenges and opportunities for early-life HIV-1 vaccines based on lessons from pediatric simian or simian-human immunodeficiency virus (SIV/SHIV) infection models and introduced the concept of early-life vaccination to protect against HIV infection in adolescence (34). Young adults aged 15 to 24 represent a high risk for HIV-1 infection and are the only population in which HIV infection continues to rise (3). Women account for two-thirds of these young adults, thereby directly linking the epidemics in young adults and pediatric HIV infections. Despite successful implementation of HIV prevention and treatment strategies worldwide, young adults have some of the lowest rates for HIV testing and adherence to antiretroviral therapy when HIV positive. To protect these young women, a vaccine would likely be required to have induced protective immunity prior to sexual debut. Yet, while pediatric vaccine coverage has steadily increased, in most low-income countries, no national vaccine programs exist for adolescents (35, 36). Dr. De Paris noted that, similarly to the PACTG 236 trial that had demonstrated the immunogenicity of an ALVAC-HIV vaccine combined with Env protein in human infants, poxvirus-based (MVA or ALVAC) SIV vaccines in infant macaques were immunogenic and modestly protective against repeated oral SIV challenges in a breastfeeding model (37). Importantly, infant macaques protected against oral SIV challenge by MVA-SIV or ALVAC-SIV vaccination as neonates were protected against rechallenge with SIV at juvenile age (37), directly supporting the concept of early-life HIV vaccination.

Discussing the distinct infant immune system, Dr. De Paris pointed out that our knowledge of immune ontogeny is steadily increasing, informing efforts to enhance vaccine immunogenicity in early life. In particular, adjuvants may be a key approach to enhance infant responses to HIV vaccines. Although the superiority of MF59 over alum in inducing durable Env-specific antibody responses in infants was documented in the early PACTG 236 trial, alum remains the most common adjuvant in pediatric vaccines. MF59 has only recently been approved for use in human adult vaccines. Dr. De Paris highlighted a study by the Precision Vaccines Laboratory, directed by Dr. Levy, in which neonatal macaques were immunized with the alum-adjuvanted pneumococcal vaccine (PCV), which is poorly immunogenic in human infants, without or with the lipidated TLR7/8 agonist 3M-052 as an additional adjuvant. Neonatal macaques receiving the PCV with 3M-052 developed antibody responses markedly earlier and at a substantially higher magnitude than infants receiving alum-adjuvanted vaccine (10). Consistent with these data, Dr. De Paris and Sallie Permar (Duke University School of Medicine, Durham, NC) recently demonstrated that, when added to HIV Env protein, 3M-052 in stable emulsion induced greater quantity and quality of antibody responses in infant macaques compared with alum or TLR4 agonist (14). These findings highlight the importance of studies testing different adjuvants in infants for their safety and efficacy in enhancing pediatric immune responses. Key for the HIV vaccine field will be to define which immune responses can provide protection and whether it is possible to elicit bNAbs by vaccination. Recent studies have documented that HIV-infected infants, despite more limited somatic hypermutation, develop bNAbs more rapidly than adults (6, 7, 38). These observations lend further impetus to studying HIV immunization in infancy to allow the development of these bNAbs and boost response prior to adolescence and sexual debut. Neonatal HIV immunization is attractive as birth is the most reliable health care contact in resource-poor countries where most HIV infections occur, and the expanded program on immunization (EPI) schedule could be amended to incorporate infant HIV immunizations. The progress of vaccine strategies to elicit bNAbs to HIV was the focus of the last session.

## PEDIATRIC HIV VACCINES—CLINICAL CANDIDATES AND IMMUNIZATION STRATEGIES

Dr. Haynes summarized the current portfolio of vaccine candidates and platforms and how these have evolved since the initiation of HIV vaccine studies over 30 years ago. A number of distinct concepts have been evaluated in clinical trials (39, 40). The failure of multiple HIV vaccine platforms to elicit highly protective immunity or broadly neutralizing antibody responses in human adults underscored the need for novel approaches that would elicit fundamentally different immunity than earlier vaccine approaches. One approach to improving the quality of the HIV vaccine responses has been the presentation of native-like envelope trimer immunogens to the immune system, which has been attempted using gp140 subunit immunogens (41), fold-on trimers (42), native flexible linked (NFL) trimers (43), DNA vaccines encoding polyvalent gp120s (44), and mRNA vaccine approaches (45–47). John P. Moore (Weill Cornell Medical College, New York, NY) shared the status of SOSIP trimer immunogens that stand out as both engaging multiple bNAbs, eliciting vaccine strain-specific neutralization, and having overcome manufacturing challenges to yield clinical-trial-ready product (48–51). Though the native-like immunogens are certainly an advance for HIV vaccine development, animal studies indicate that limited presentation of the correctly folded native-like immunogen to a mature adult immune system is unlikely to yield broad and protective HIV-specific immunity that will be required for a highly effective vaccine.

The B cell lineage design approach to HIV vaccine development emerged as a reverse vaccinology strategy guided by isolation of a collection of broadly neutralizing monoclonal antibodies from a subset of HIV-infected individuals who developed broad neutralizing responses. The preclinical HIV vaccine pipeline has further diversified to include candidate vaccines designed to specifically engage germ line immunoglobulin variable genes identified to develop into bNAb lineages in a subset of HIV-infected individuals. The next-generation HIV vaccine candidates include full-length single chain (52), engineered outer domain gp120 CD4 binding site (eOD-GT8 CD4bs) immunogen (53), 426c deglycosylated heptamer (54), variable loop 3 (V3) glycopeptides (55), Env fusion peptide (56), and membrane-proximal external region liposomes (57). In addition, a suite of gp120 immunogens, CH505 Env Sequences, isolated from an HIV-infected patient who developed bNAb responses at critical branches in the HIV-specific B cell lineage development, have been developed as potential immunogens to guide B cell induction and evolution down the same pathway that successfully resulted in bNAb responses during infection (58). These sequential gp120 immunogens are currently being assessed for safety and immunogenicity in adults (HVTN115), specifically looking for whether unmutated common ancestors of CD4 binding site-specific bNAb variable gene lineages can be induced by these low-affinity gp120 ligands. Finally, combining the native-like immunogen and germ line-targeting approaches, SOSIP trimers are currently being designed to bind to unmutated ancestors of bNAb immunoglobulin genes (59).

Due to the limited diversity of the B cell repertoire in infants and the long window of time that pediatric immunization offers prior to sexual debut and risk of horizontal HIV acquisition, early-life immunization with these types of B-cell-lineage-targeting HIV vaccines could be more fruitful than immunizing in adulthood (34). As infected individuals typically do not develop bNAb activity in plasma until years into their infection, immunization strategies to achieve broad and potent antibodies may also require years of time and multiple immunogen exposures. Moreover, infants demonstrate distinct B cell tolerance regulation compared to adults (60–62), which could be beneficial to the strategy of engaging and positively selecting B cells expressing specific germ line immunoglobulin gene sequences. Thus, testing the next generation of candidate HIV vaccines in infants may be critical to exploring their full potential.

Dr. Permar discussed the importance of maternal antibodies in the protection of infants against mother-to-child transmission (MTCT) and their possible role in infant HIV vaccination. In the absence of intervention, >60% of HIV-exposed infants will be protected against HIV acquisition, suggesting the presence of naturally protective factors. As maternal antibody is transferred to infants via the placenta during the latter half of pregnancy, one possibility for natural protection is partial protection from preexisting passive humoral immunity. Yet, the impact of maternal antibody responses on MTCT in HIV-exposed infants has been controversial, with studies of various cohorts demonstrating apparently conflicting evidence of maternal humoral immune responses being associated with either a protective effect (63, 64) or a lack of protective effect (65) on autologous infant virus acquisition. One recent study in an untreated, U.S.-based cohort associated reduced risk of perinatal transmission with the presence of maternal V3 and CD4 binding site (CD4bs)-specific weakly neutralizing IgG levels (66). Interestingly, these maternal weakly neutralizing antibodies against the V3 and CD4bs demonstrated activity against autologous maternal viruses, suggesting that enhancing these maternal responses, achievable with current HIV vaccine candidates, could be a strategy to further reduce MTCT. As discussed earlier by Dr. Overbaugh, infant transmitted variants are more resistant to paired maternal plasma neutralization than nontransmitted maternal variants, suggesting that weak neutralization of cocirculating viruses is a potential mechanism for their protective effects. Manish Sagar (Boston University School of Medicine, Boston, MA) emphasized that some strategies to enhance maternal NAb responses may be associated with higher risk of HIV transmission to infants (67), which may be a result of selection pressure on transmitted variants. Thus, the role of maternal antibodies in their ability to neutralize cocirculating viruses and their role in selection of infant transmitted/founder virus strains remains to be further examined to determine if strategies to enhance maternal virus-specific antibodies during pregnancy would be safe and effective.

A second important question for infant vaccination is the impact of preexisting maternal or passively administered antibodies on active vaccine immunogenicity. Active immunization of HIV-exposed infants to further reduce the risk of postnatal infection would be performed in the setting of placentally transferred maternal antibody. Of note, early studies of gp120 subunit immunization of HIV-exposed newborns indicated that infants develop robust antibody responses in the setting of preexisting maternal antibodies. Moreover, maternal antibody levels present at birth in the infant did not predict the specificity or magnitude of the infant’s active vaccine response (20). Panelist Ann Hessell (Oregon Health and Science University, Portland, OR) discussed the impact of passive immunization with bNAbs as a viable short-term protection strategy for frequent exposure to HIV-1 via breastfeeding. By analogy, a combination of passive and active HIV immunization in infancy, mimicking the highly successful perinatal hepatitis B immunization strategy, may be effective. Interestingly, the presence of preexisting neutralizing antibodies has improved the neutralization response to HIV-1 infection in both humans and NHPs (68, 69). Finally, the administration of a passive bNAb at delivery may block neonatal infections when administered within 24 h (33), suggesting that delivery of bNAbs in the delivery room may abort infections that were initiated from exposure during delivery. Thus, the combined passive and active perinatal HIV vaccine approach could achieve both protection for the infant against perinatal and postpartum infection and enhanced long-term immunity. The early HIV vaccine studies in the PACTG 230 and 236 trials and a recent study in infant rhesus macaques support the feasibility of active infant immunization in the presence of passively acquired HIV-specific antibodies. Nevertheless, further studies are needed to thoroughly define the impact of preexisting maternal or passively infused antibodies on the active response to the HIV vaccination.

## SUMMARY

The consultation provided an opportunity for experts in HIV vaccines and infant immunity to address the scientific rationale for development and testing of early-life HIV vaccines. The panel emphasized that despite the overwhelming success of preventing mother-to-child transmission of HIV, infants are still vulnerable to the virus, especially during the breastfeeding period. A strong precedent for prevention of infections by early-life immunization and passive/active infant immunization regimens, such as the hepatitis B vaccine, encourages the field to explore if new and more effective HIV vaccine strategies for this age group to elicit optimal immune responses for durable immunity are feasible. Dynamic early-life immune ontogeny as well as prior infant vaccine trials suggests that this concept may have merit to test candidate HIV vaccines designed to induce broad and durable protection ahead of sexual debut.

## KEY POINTS AND QUESTIONS

- Prior infant vaccine studies support the concept that the pediatric population offers unique opportunities to test candidate HIV vaccines and inform objectives and deliverables of future infant HIV vaccine clinical immunogenicity trials.
- What will the scientific criteria be for selection and testing of candidate HIV vaccine immunogens in infants?
- What are the challenges of conducting HIV vaccination trials in pediatric population, and how can the clinical developmental plan for infant HIV vaccines be pursued in parallel with that in adults?
- What are the unique opportunities for augmenting immunity in infants? How will the distinct early-life immune system, including age-specific adjuvant-immunogen synergy, passively acquired maternal antibodies, and developing gut microbiota, shape responses to pediatric HIV vaccines?
- Does the current panel of assays, technologies, tools, and models accurately profile immune responses to HIV vaccines in infants to inform rational design of infant HIV vaccine candidates?
- What is the impact of the distinct immune system and passively acquired maternal anti-HIV antibodies of HIV-exposed uninfected infants on early-life vaccine immunogenicity?
